# Supplementary material for: Reconstitution of bacterial autotransporter assembly using purified components
Source: eLife. 2014 Sep 2;3:e04234. doi: 10.7554/eLife.04234 (PMC4174580; doi:10.7554/eLife.04234)
Supplement: Supplementary file 1. — Kinetic analysis of EspP assembly in vitro. DOI: http://dx.doi.org/10.7554/eLife.04234.019 [file elife04234s001.docx]

**File Supplement 1. Kinetic analysis of EspP assembly in vitro**

**Proteolytic maturation data fit to single exponential kinetics:**

**EspP derivative k (min^-1^) R t_½_ (min)**

EspP(46+β) 0.332 0.975 2.1

EspP(46+β)G1066A 0.220 0.980 3.2

EspP(HA-251+β) 0.338 0.965 2.1

EspP(HA-251+β)G1066A 0.216 0.971 3.2

EspP(HA-714+β   2.7

EspP(HA-714+β586TEV 0.198 0.978 3.5

**Proteolytic maturation data fit to lag-phase kinetics:**

**EspP derivative k_1_ (min^-1^) k_2_ (min^-1^) R t_½_ 1 (min) t_½_ 2 (min)**

EspP(46+β) 0.806 0.785 0.993 0.86 0.88

EspP(46+β)G1066A 0.572 0.581 0.994 1.2 1.2

EspP(HA-251+β) 0.799 0.800 0.989 0.87 0.87

EspP(HA-251+β)G1066A 0.570 0.562 0.990 1.2 1.2

EspP(HA-714+β) 0.639 0.642 0.987 1.1 1.1

EspP(HA-714+β)586TEV 0.522 0.528 0.996 1.3 1.3
