# Supplementary material for: Reconstitution of bacterial autotransporter assembly using purified components
Source: eLife. 2014 Sep 2;3:e04234. doi: 10.7554/eLife.04234 (PMC4174580; doi:10.7554/eLife.04234)
Supplement: Supplementary file 2. — Oligonucleotides used in this study. DOI: http://dx.doi.org/10.7554/eLife.04234.020 [file elife04234s002.docx]

Supplementary File 2. Oligonucleotides used in this study

pTRC/Nde 5’-CACACAGGAAACAGCATATGGAATTCGAGCTCGG-3’

BamA(+) 5’-GATATACATATGGTTAGGAAGAACGCATAATAACG-3’

BamA(-) 5’-CTTATTAGGATCCCACTTACCAGGTTTTACCGATG-3’

BamB(+) 5’-TAATAATTTGTAGATCTGAGAGGGACCCGATGC-3’

BamB(-) 5’-GTGAACGACGGATCCGATTAACGTGTAATAGAGTAC-3’

BamC(+) 5’-CTTAAGCATGCCGGTTTGCTGTAGATCTTAGGGAG-3’

BamC(-) 5’-GCCGGATGATTCCAGCCCTGGATCCTTACTTGCT-3’

BamD(+) 5’-CTCCCGTATTAGATCTTGAGGAAAGTCAAAACG-3’

BamD(-) 5’-GGCTGCCGTTTTGTGTTTCAGGATCCTGTTATG-3’

BamE-His_8_(+) 5’-AGCCACGTACTGCTCGGATCCCGAAAAGGA-3’

BamE-His_8_(-) 5’-CTGGAGCGCTCAGATCTTAGTGGTGATGATGGTGGTGATGATGACC

ACCGTTACCACTCAGCGCAGGTTTGTTATC-3’

BamA.Eag 5’-CCACCGTATACGCGGCCGAAGGGTTCGTAGTG-3’

BamAHis(+) 5’-pGGCCCATCACCATCACCACCATGGCGC-3’

BamAHis(-) 5’-pGGCCGCGCCATGGTGGTGATGGTGATG-3’

EspP (-) 5’-TTTGAATACGGATCCTCAGAACGAGTAACGGAAATTAGC-3’

EspP(46+β) 5’-ACGGTAGCAAACAAGCATATGGCAAACAAGGAAGCAACCCGG-3’

EspP(51+β) 5’-ACGGTAGCAAACAAGCATATGGGCTA-3’

EspP(72+β) 5’-ACGGTAGCAAACAAGCATATGGATGTAACGCCGGTCATTACA-3’

EspP(96+β) 5’-ACGGTAGCAAACAAGCATATGATTGAACTGGTAAGCGCGCCA-3’

EspP(218+β) 5’-GATGAAAGCGACCTCTCGCATATGTCTGAC-3’

EspP(271+β) 5’-CAGGCAATCCCGACAAAGACCATATGTTCGAGCC-3’

EspP(324+β) 5’-GCAGAAGACAGCACTATCGTTATTAAACATATGGCAAAAGC-3’

EspP(524+β) 5’-CCCTGAATAATACTGCTGACCATATGTATCACGG-3’

EspP(734+β) 5’-GGAACCAGACAACCATTGACAACCATATGAACAAGT-3’

RD_1512-1619_(+) 5’-GTTCGCCAGCCCCATGGACGAACCC-3’

RD_1512-1619_(-) 5’-CTGGCGCGCGAACCACATATGGTCC-3’

RD_1457-1677_(+) 5’-CATATTCTTTTGCGTCCATGGGCGAAGCCGGTGAC-3’

RD_1457-1677_(-) 5’-ATAGGATCCGTCCATATGCTGCGCCATTGCCTCG-3’

HA tag(+) 5’-pCATGGGTTATCCGTACGATGTTCCAGATTACGCAAGCCTGGG-3’

HA tag(-) 5’-pTACCCAGGCTTGCGTAATCTGGAACATCGTACGGATAACC-3’
